# The Influence of Light on Olive (*Olea europaea* L.) Fruit Development Is Cultivar Dependent

**DOI:** 10.3389/fpls.2019.00385

**Published:** 2019-03-27

**Authors:** Lara Reale, Luigi Nasini, Martina Cerri, Luca Regni, Francesco Ferranti, Primo Proietti

**Affiliations:** Department of Agricultural, Food and Environmental Sciences, University of Perugia, Perugia, Italy

**Keywords:** cyto-histological observations, endocarp, fruit oil content, fruit shading, mesocarp

## Abstract

In olive, the response to environmental conditions, such as light availability, is under genetic control and requires a combination of biochemical and physiological events. We investigated the effect of irradiance in fruit development in two Italian cultivars, Leccino and Frantoio. Morphological and cyto-histological analyses, as well as water and oil content determination, were carried out in fruits exposed to a different light regime (named as light and shade fruits). Results demonstrated that the influence of light availability on fruit development depends on the cultivar. In Leccino, the fresh and the dry weight, the percentage of dry matter, the kernel and fruit diameter, the mesocarp thickness and the mesocarp cell size were higher in the light exposed fruits than in the ones grown in the shade. In Frantoio, differences between light and shade fruits were observed only at 140 DAF (Days After Flowering) and only in the kernel and fruit diameter and in the dry and fresh weight, which were higher in the light exposed fruits. Leccino, therefore, showed a greater sensitivity to the light availability. This may be related to the observed delay in the endocarp lignification as compared to the Frantoio cultivar. In each cultivar, moreover, shade and light fruits did not show differences in the timing of cell differentiation. Finally, the investigation of oil storage carried out in cyto-histological studies demonstrated that differences in oil content between fruit subjected to different light regimes correlated with the number of oil containing cells, rather than the oil content per cell. A different behaviour was observed in the two cultivars: in Leccino, the mesocarp cell size was almost twice of Frantoio, while oil drops were only 30% larger; therefore, the percentage of cell volume occupied by the oil drops was lower in Leccino than in Frantoio. The chemical analysis confirmed this observation.

## Introduction

The sunlight use efficiency (i.e., dry matter formed per unit of photosynthetically active radiation absorbed) has long been investigated to improve quantity and quality of fruit harvesting. Optimisation of the sunlight use has been obtained in orchards systems by the improvements in some cultural choices, such as orchard design, training system, and pruning, directed toward the upgrading of photosynthetically active radiation (PAR) interception and distribution within the canopy (Jackson, 1980; Palmer, 1989; Proietti and Palliotti, 1997; Proietti, 1998; Corelli-Grappadelli, 2003; Corelli-Grappadelli and Lakso, 2007). In olive production, there is extensive knowledge of the importance of the sunlight irradiance (Connor et al., 2009, 2016; Proietti et al., 2012; Cherbiy-Hoffmann et al., 2013; Trentacoste et al., 2016; Caruso et al., 2017). Olive fruits grown under well lighted conditions are heavier than those grown at low light intensity, have a higher percentage of oil, and a lower water content (Ortega Nieto, 1962; Gómez-del-Campo and García, 2012; Proietti et al., 2012; Benelli et al., 2016; Caruso et al., 2017). Moreover, the oils obtained from olives grown under well lighted conditions had higher phenol content and better sensorial characteristics than those extracted from less lighted fruits (Proietti et al., 2012). Many biochemical and physiological events contribute to olive fruit development and they are strongly influenced by several environmental conditions and genetic background (Proietti and Tombesi, 1996). At the histological level, olive fruit is characterised by a fleshy mesocarp (pulp) and a hard endocarp (pit), both contributing to fruit growth following different morphogenic patterns (Gucci et al., 2009). Fruit development, as reported by Conde et al. (2008), lasts for 4–5 months and is characterised by 5 stages: (I) fertilisation and fruit set, during which the early mitotic activity promotes embryo’s growth; (II) seed development, the first period of rapid fruit growth, characterised by the little mesocarp development and the intense growth of endocarp as a result of division and expansion cell activity (seed/pit); (III) pit hardening, due to differentiation of endocarp cells, which stop dividing and become sclerified; (IV) mesocarp development, the second major period of fruit growth, mainly characterised by the expansion of parenchima cells in a radial direction and by an intense oil accumulation, and (V) ripening, that represents the last stage of development, in which the biochemical and physiological changes lead to an acquiring of a characteristic appearance, texture, flavour and aroma. In olive, ripening starts when the fruit colour changes from hard green to yellow-green (Conde et al., 2008), as a result of chlorophyll degradation.

Potential fruit size is genetically regulated (Padula et al., 2008) and may greatly differ between olive cultivars (Barranco, 1999). These differences are mostly determined by cell number, although cell expansion is the great driving force for the growth of the fruit (Rapoport et al., 2004; Hammami et al., 2011). Both these processes (division and expansion) contribute to fruit growth but in different percentages and at different times. As reported by Hammami et al. (2011), in mesocarp, which is the largest and economically most important olive tissue, growth occurred mainly due to cell division during the first 8 weeks after bloom; in this period “65% of final mesocarp cell number and 25% final cell size were reached.” However, in the successive phases of fruit development (8–32 weeks) growth was mainly related to cell expansion and a 75% increase in cell size was achieved.

Environmental conditions can determine fruit size by affecting mitotic activity, cell expansion or both (Denne, 1960; Bergh, 1985; Costagli et al., 2003; Gucci et al., 2009). Trentacoste et al. (2016) investigated the influence of irradiance in the olive fruit development; they demonstrated that fruit and mesocarp weight and oil content increase from the base to the top of the canopy, suggesting a linear relation to irradiance. Otherwise, endocarp weight and composition are little affected by light availability. Different assimilation levels influence the rate of endocarp development, but not its final size or composition, showing the strong sink activity of this tissue. Differences in mesocarp weight among canopy position was not influenced by fruit number, indicating that the sink competition was not a limiting factor (Trentacoste et al., 2016). While the role of division and expansion in fruit development has long been investigated, their relationship with assimilate (e.g., oil) storage is only beginning to be explored. Trentacoste et al. (2016) showed that mesocarp composition was influenced by cell number and size: 51 and 67% of oil content variation among fruit position was due to changes in mesocarp cell number and mesocarp cell size, respectively. Cell number and the rate of cell expansion during the last phases of fruit growth appears to play an important role in import of assimilate into the olive (Gillaspy et al., 1993; Génard et al., 1999) but also in other fruit as tomato (Bertin et al., 2003). Mesocarp composition is the most important commercial criterion in olive and is largely influenced by genotypes, environment and management, but the association between expansion, oil storage and cell processes, during the mesocarp development is yet still poorly understood. In this study two olive cultivars were considered: Frantoio and Leccino. Cultivar Frantoio is a widely spread Italian variety. It is a medium vigour tree, it has a high lateral development and a medium density of foliage. The fruit branches are hanging, and the fruits ripening is scalar and late. It is highly valued for its high and consistent productivity, and for its ability to adapt to different environmental conditions; it’s, however, sensitive to winter cold temperature. It produces medium-size fruit with medium to high oil content. The oil produced is greatly appreciated for its excellent organoleptic characteristics and stability. Cultivar Leccino is vigorous, able to easily adapt to different soil conditions and highly tolerant to cold temperatures. It has a high lateral development and a high density of foliage. The fruit branches are hanging. It is valued for its early onset of bearing and its high and consistent productivity. It has medium-size and very early ripening fruits, with low retention force thus making easy to harvest. It has low oil content (Barranco et al., 2010).

In this context, the aims of this work were to: (1) study the influence of light availability on olive fruit development; (2) investigate oil storage in mesocarp cells during the different phases of fruit development and at different light conditions; (3) compare the behaviour of two Italian cultivars to verify the presence of eventual differences.

## Materials and Methods

### Plant Material

The trial was carried out in 2011 in central Italy, Deruta (PG) latitude 43° North, in a non-irrigated, slightly sloping olive grove (350 m a.s.l.). The climate of the area is characterised by mild winters and warm, dry summers, with an annual average rainfall of 850 mm, mainly distributed in autumn and winter. The soil was medium textured and was kept under clean cultivation throughout the entire growing season. The 20-year-old trees from cultivars Frantoio and Leccino were spaced 5 × 5 m and trained to the vase system.

Ten uniform trees per cultivar having a fruit load at harvest of about 20 ± 1.6 kg of olives per tree were selected. The trees were about 4 ± 0.35 m high, with a canopy of 4.5 ± 0.30 m in diameter and 3.5 ± 0.25 m in height. Two hundred fruiting branches (20 per tree) were selected in June; in these branches, during the day at the beginning of the experimentation, the light availability was measured in the fruiting portion (average of three measurements for each branch), using a Licor Quantum/Radiometer/Photometer – 185B. Measurements were taken on south external portion (at about 150 cm in height from the ground) and in the centre (at about 150 cm in height from the ground and 200 cm from the edge) of the canopy for lighted and shaded branches, respectively (Table 1). Data collected showed, at 11:00 am, in shade branches a light availability of around 400 μmol (photon) m^-2^ s^-1^; in these conditions net photosynthesis in olive is half that at photon saturation, i.e., about 1000 μmol (photon) m^-2^ s^-1^ (Proietti and Famiani, 2002), that is the value observed in our light branches. Fruits for all the determinations and observations were sampled from the south external and internal portion of the canopy. In particular, for the morphological observations, 30 shade and light fruits, (from the middle part of the labelled shade and light branches, respectively) collected at 50, 80, and 140 DAF (Days After Flowering), were considered. The full bloom (50% of open flowers) occurred at the same time (1st–3rd June) for the two cultivars.

**Table 1:** Average light availability ± standard error at different hours (15th June) in the south external portions of the canopy (at about 150 cm in height from the ground) and in the centre (at about 150 cm in height from the ground and 200 cm from the edge) where light/shade fruits were taken in Frantoio and Leccino cultivars.

Hour of the day	Frantoio		Leccino		Full sun light
	Light	Shade	Light	Shade	
		
	PPFD (μmol m^-2^s^-1^)				
8:00	303 ± 32	182 ± 14	290 ± 33	162 ± 19	515 ± 38
11:00	1030 ± 53	407 ± 42	984 ± 54	397 ± 66	1391 ± 74
14:00	951 ± 48	369 ± 31	938 ± 50	315 ± 33	1326 ± 62
17:00	605 ± 34	313 ± 22	585 ± 31	309 ± 29	828 ± 36


*The light availability at the full sun near the considered trees is also reported.*

### Morphological Observations

The selected dates for morphological observations correspond to three of the five stages of fruit development: epicarp and mesocarp differentiation (50 DAF, stage II), pit hardening (80 DAF, stage III), and mesocarp development (140 DAF, stage IV) (Conde et al., 2008). Fresh weight and diameter (by calliper) of fruits and kernel were detected. To determine the dry weight and the percentage of the dry matter, 30 shade and light olive fruit for each cultivar, collected at 140 DAF, were weighted before and after being oven dried at low heat (95°C) to constant weight.

### Cyto-Histological and Morphometric Observations

To conduct cyto-histological observations, 30 shade and light fruits were collected at 14, 21, 50, 80, and 140 DAF. The stages were confirmed, before image analysis, by observation of semi-thin sections under a light microscope, according to Conde et al. (2008). Fruit portions were fixed in 3% (w/v) glutaraldehyde in 0.075 M cacodylate buffer, pH 7.2, for 10 h. The samples were then washed three times for 7 min in 0.075 M cacodylate buffer, pH 7.2, post-fixed in 1% (w/v) OsO_4_ in the same buffer for 1 h, dehydrated in increasing concentrations of ethanol and, at the last, embedded in epoxy resin (Epon, 2-dodecenylsuccinic anhydride and methylnadic anhydride mixture) (Reale et al., 2016, with modifications). Semi-thin sections (1–2 μm), obtained with an ultramicrotome (OmU2, Reichert, Heidelberg, Germany) equipped with a glass blade, were stained with toluidine blue or by Periodic Acid Schiff’s reaction (O’Brien and McCully, 1981) and observed under a light microscope (BX53; Olympus, Tokyo, Japan). For the Periodic Acid Schiff’s reaction, semi-thin sections were treated with 0.5% periodic acid for 30 min at 40°C, washed with tap and demineralised water, and covered with Schiff’s reagent for 15 min. Sections were then washed rapidly with tap water, washed two times for 3 min with SO_2_ water, and washed two times for 10 min with demineralised water. Sections were also counter-stained for protein with 1% (w/v) amido black in 7% acetic acid. The presence of proteins was indicated by a blue colour, while starch grains appeared magenta. For the toluidine blue staining, semi-thin sections were covered with 0.5% (w/v) toluidine blue in 2% NaHCO_3_ buffer. Toluidine blue has a high affinity for acidic tissue components and stains nucleic acids blue and polysaccharides purple. For each stage, 50 semi-thin sections obtained from at least 10 fruits, were observed under a light microscope (BX53; Olympus, Tokyo, Japan) equipped with image analysis software (CellSens Standard; Olympus, Tokyo, Japan). In 30 of these sections collected at 50, 80, and 140 DAF (10 semi-thin sections for each date), mesophyll thickness, transversal area of 10 parenchyma cells and 10 oil drops were also measured. In the text, we refer to “cell transversal area” as “cell size.” The thin sections (0.08 μm) obtained with an ultramicrotome (OmU2, Reichert, Heidelberg, Germany) equipped with a diamond blade, were mounted on uncoated copper grids (200 mesh) and contrasted by adding uranyl acetate and an aqueous solution of lead nitrate. Observations were carried out with a transmission electron microscope (TEM 400 T; Philips Electron Optics – FEI Company, Hillsboro, OR, United States).

### Oil and Water Content

At the harvesting, 20th November (170 DAF), 40 shade and light olive fruits for each cultivar were collected and oil and water content were determined in 3 replicates of the same weight, using the “SpectraAlyzer ZEUTEC” – NIR: Near Infra-Red (Cayuela et al., 2009). The near infrared radiation covers the spectrum range electromagnetic between 780 and 2500 nm. NIR spectroscopy consists in irradiating the product and measuring the radiation, reflected or transmitted. During the penetration of the radiation in the product, its spectral characteristics vary through the wavelength, both for the dispersion (scattering) of light and for the absorption processes. This variation depends on the chemical composition of the product as well as the properties of the scattering, they are variables related to the microstructure of the matrix (Nicolai et al., 2007; Ruiz-Altisent et al., 2010).

### Statistical Analysis

The statistical analysis was conducted in R environment (R Core Team, 2015). Values were compared by a two-way non-parametric ANOVA and *t*-test analysis and *P* ≤ 0.05 was considered significant. Data are reported as average ± standard error or standard deviation.

## Results

### Morphological Observations

**FIGURE 1:**
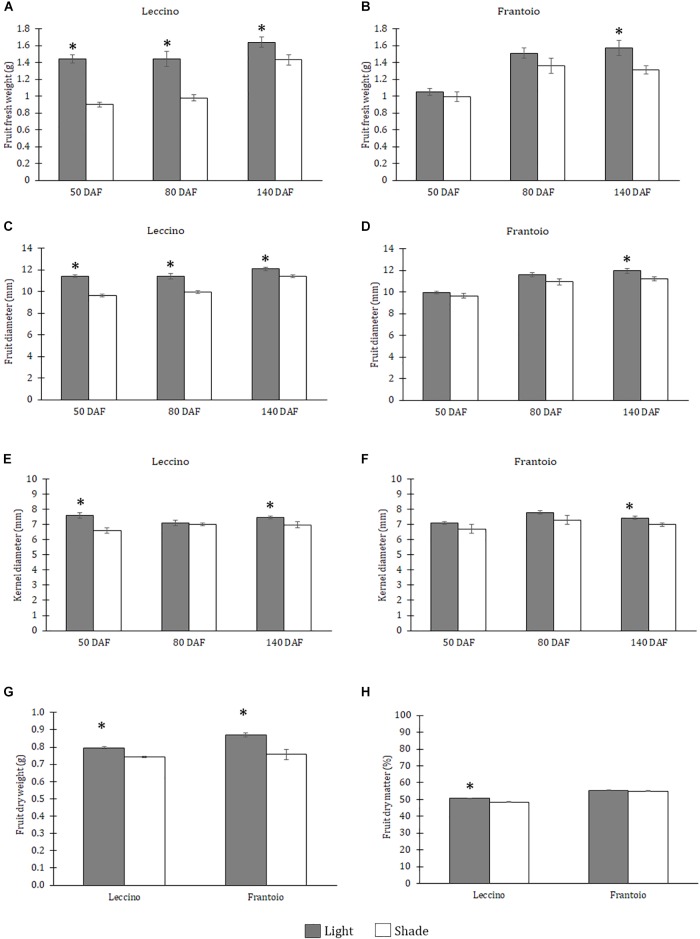
Fruit fresh weight **(A,B)**, fruit diameter **(C,D)** and kernel diameter **(E,F)** in Leccino **(A,C,E)** and Frantoio **(B,D,F)** shade and light fruits at different developmental stages. Dry weight **(G)** and dry matter **(H)** in Leccino and Frantoio shade and light fruits at 140 DAF. Data are reported as means ± standard errors per cultivar (Leccino and Frantoio) and light environment (light and shade). The asterisks indicate significant differences (*t*-test) between treatments after Analysis of Variance within each date of sampling (*P* < 0.05).

In Leccino, at all dates, significant differences in fresh weight and diameter were observed between shade and light fruits: shade fruit had a lower fresh weight (Figure 1A) and a lower diameter (Figure 1C) than the light ones. In Frantoio, shade fruit had a lower fresh weight (Figure 1B) and a lower diameter (Figure 1D) than the light ones, but differences were significant only at 140 DAF. For both these parameters differences between cultivars were significant only at 50 and 80 DAF (Supplementary Table S1) and the interaction between cultivar and light condition was significant only at 50 DAF (Supplementary Table S1). With respect to the kernel diameter, in Leccino, significant differences were observed at 50 and 140 DAF, when light fruit showed larger kernel diameter than shade ones (Figure 1E). In Frantoio, at all dates, shade fruit had a smaller kernel diameter than the light ones, but differences were significant only at 140 DAF (Figure 1F). For this parameter, slight differences were observed between cultivars only at 80 DAF and no significant interaction was observed between light and cultivar (Supplementary Table S1).The dry weight of light fruits was higher than the shade ones in both cultivars (Figure 1G); differences were also observed between cultivars, without interaction with light (Supplementary Table S1).The percentage of dry matter was not different in light and shade fruits of Frantoio, while this percentage was significantly higher in Leccino light fruit than shade ones; indeed, a significant interaction was observed between cultivar and light condition (Figure 1H and Supplementary Table S1). Differences were observed between cultivars: both light and shade fruit of Frantoio showed higher percentage of dry matter than the Leccino ones (Supplementary Table S1).

### Cyto-Histological and Morphometric Observations

#### 14 DAF

**FIGURE 2:**
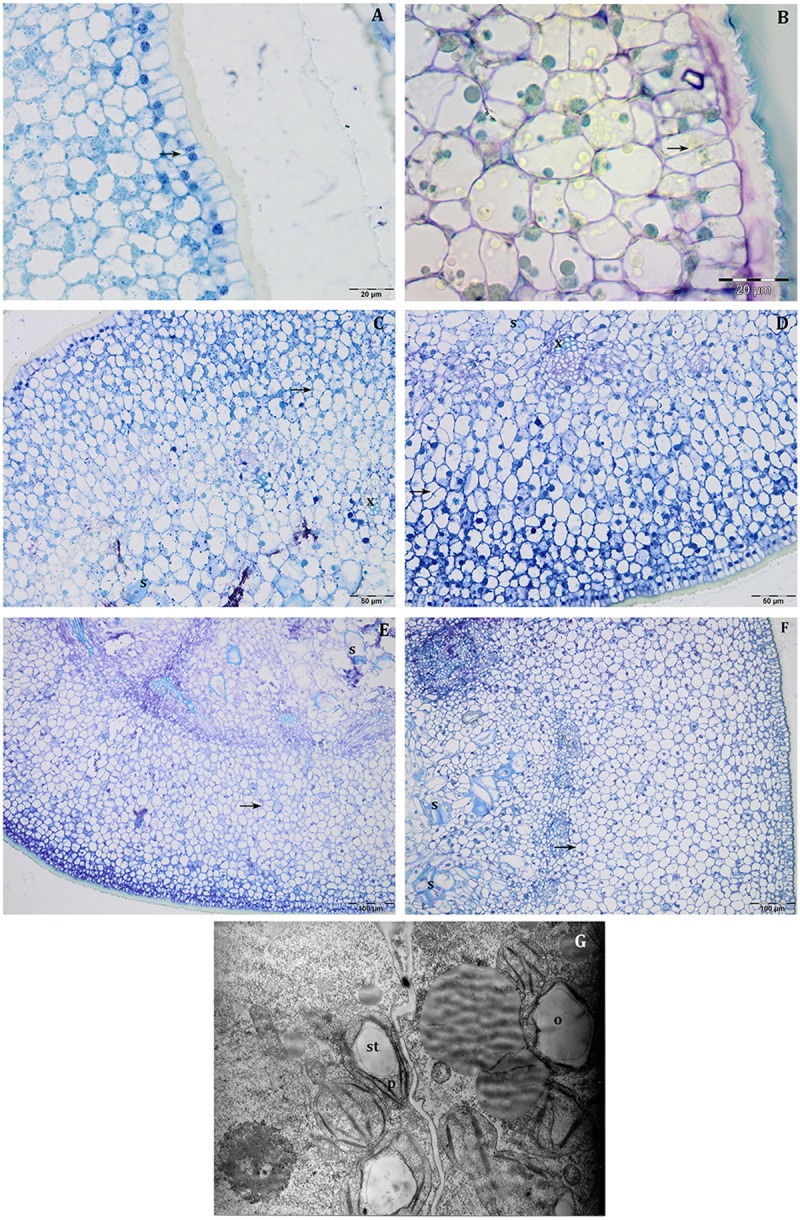
Semi-thin sections at 14 DAF **(A–D)** and 21 DAF **(E–G)** of Leccino **(A,C,E)** and Frantoio **(B,D,F)** olive fruits. At 14 DAF, in the external epidermis, anticlinal divisions (black arrow) were detected **(A,B)**, while, in the inner portion of pericarp **(C,D)**, individual sclerified stone cells (s) and xylem cells (x) were differentiated. At 21 DAF mitotic divisions were yet evident (**E,F**, black arrows), while in the endocarp sclerified stone cells (s) were detected more frequently in Frantoio **(F)** than in Leccino fruits **(E)**. **(B)** is stained with PAS reaction, while the other figures are stained with toluidine blue. **(G)** Thin section of pericarp of Frantoio light fruit at 21 DAF: the presence of primary starch (st) in the plastid (p), and the oil drops (o) were evident.

At this stage, the histological structure of the young pericarp was very similar to a leaf; the external epidermis is covered by a thick cuticle and is characterised by the presence of anticlinal divisions (Figure 2A,B). Mitotic activity was also observed in the cells that will differentiate the mesocarp and the endocarp (Figure 2C,D). In the inner portion of the pericarp, scattered individual sclerified stone cells were observed throughout the principally parenchyma cells (Figure 2C,D). In the vascular bundle, it is already possible to distinguish the first xylem cells (Figure 2C,D). Sphaerosomes were not evident, and neither starch grains were observed.

#### 21 DAF

The histological structure of the mesocarp was almost the same as that observed at 14 DAF; mitotic divisions were yet evident (Figure 2E,F). In the endocarp, groups of two or four sclerified stone cells were detected (Figure 2E,F). Sclereid cells appeared more frequent in Frantoio (Figure 2F) than in Leccino fruit (Figure 2E), suggesting the precocious endocarp lignification in Frantoio. In the semi-thin sections, the presence of starch grains or oil drops was not evident, while TEM observations demonstrated the presence of little starch grains inside the plastids and the first oil drops (Figure 2G).

#### 50 DAF

**FIGURE 3:**
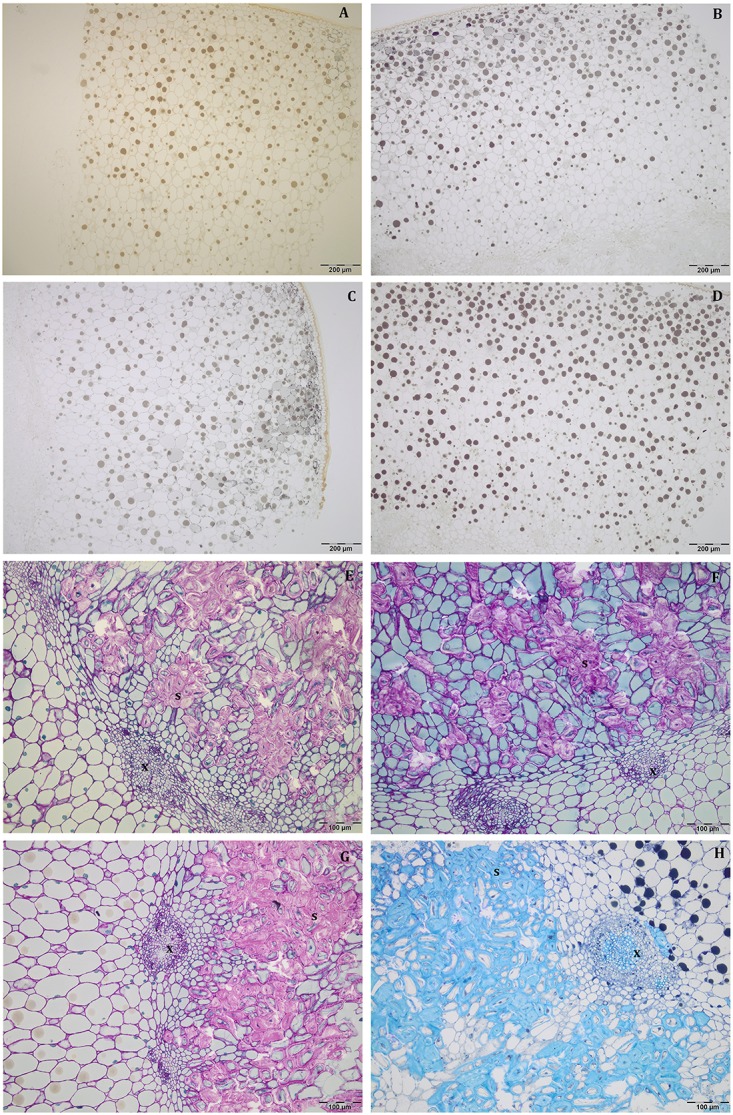
Semi-thin sections at 50 DAF of Leccino **(A,C,E,G)** and Frantoio **(B,D,F,H)** fruits in light **(A,B,E,F)**, and shade **(C,D,G,H)** conditions. Oil drops were evident (as little dark spheres) in some mesocarp cells and the endocarp was occupied by sclerified clusters **(E–H)**. **(A–D)** are stained with osmium, while **(E–G)** are stained with PAS reaction and **(H)** is stained with toluidine blue.

**FIGURE 4:**
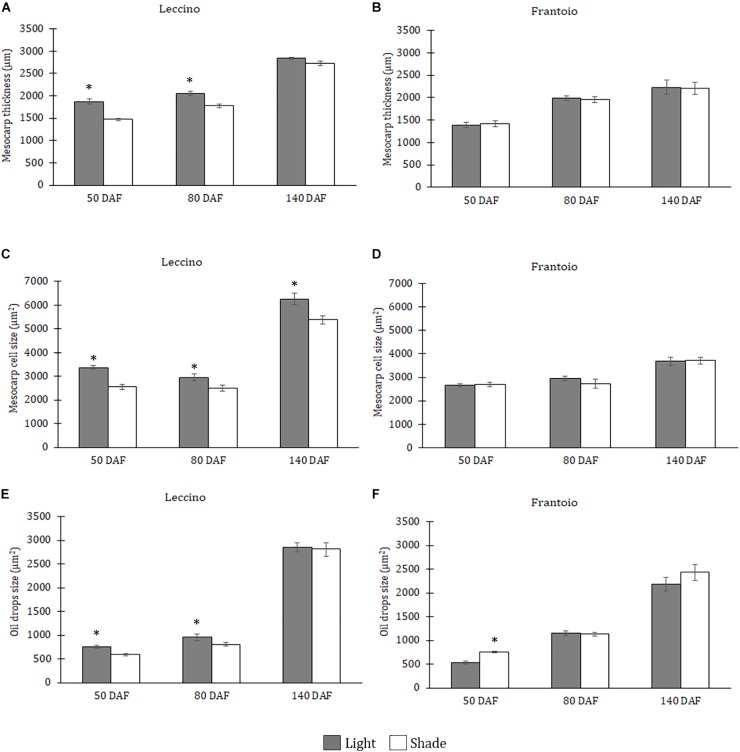
Mesocarp thickness **(A,B)**, size of mesocarp cells **(C,D)** and oil drops **(E,F)** in Leccino **(A,C,E)** and Frantoio **(B,D,F)** shade and light fruits at different developmental stages. Data are reported as means ± standard errors per cultivar (Leccino and Frantoio) and light environment (light and shade). The asterisks indicate significant differences (*t*-test) between treatments after Analysis of Variance within each date of sampling (*P* < 0.05).

At this stage, the mesocarp thickness and cell size of light fruits were significantly higher than shade ones in Leccino (Figure 4A,C), but no differences were observed in Frantoio (Figure 4B,D). For both parameters, statistically significant differences were observed also between Frantoio and Leccino (Supplementary Table S1), with a significant interaction between light and cultivar. In both cultivars and light conditions, oil drops were evident in the mesocarp by light microscope observations (Figure 3A–D), but were detected only in some cells of the mesocarp, especially in the external portion; they appeared roundish and occupied only a small fraction of the cell lumen (Figure 3A–D). The size of the oil drops at 50 DAF in Leccino was larger in light fruits compared to shade ones (Figure 4E), whereas the opposite was observed in Frantoio (Figure 4F). The interaction between light and cultivar was statistically significant (Supplementary Table S1). The endocarp area appeared to be characterised by a high number of sclerified clusters, composed of many sclereids that showed a different shape and thickening of the lignified secondary walls (Figure 3E–H). At this stage, differences between Frantoio and Leccino in the endocarp differentiation were no longer appreciable.

#### 80 DAF

**FIGURE 5:**
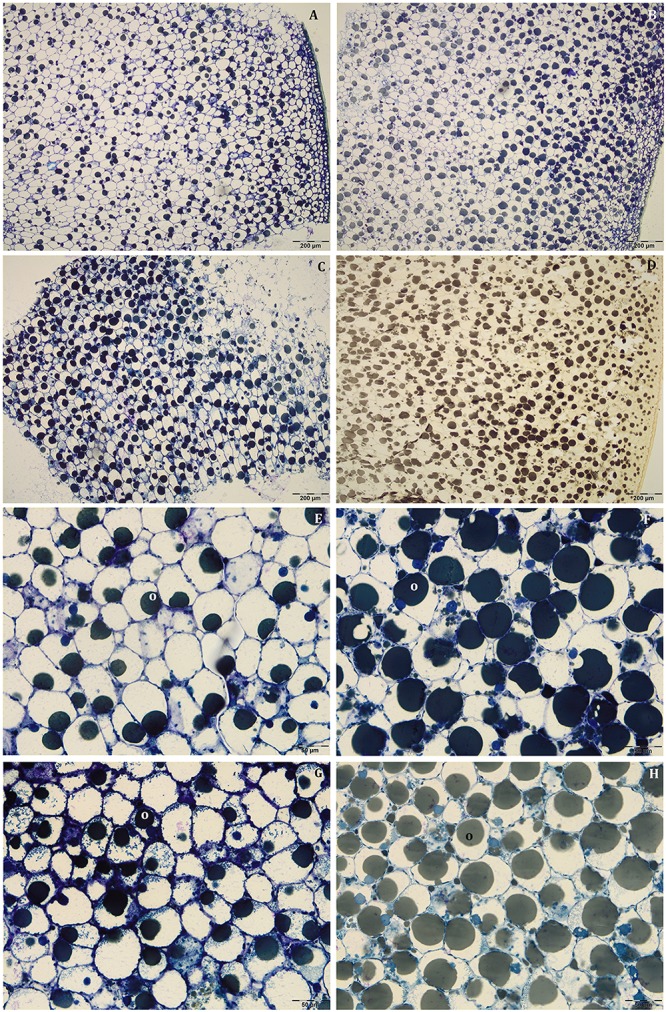
Semi-thin sections at 80 DAF of Leccino **(A,C,E,G)** and Frantoio **(B,D,F,H)** fruits in light **(A,B,E,F)**, and shade **(C,D,G,H)** conditions. Oil drops were uniformly distributed in the mesocarp **(A–D)** and occupied a larger fraction of the cell volume in Frantoio **(F,H)** than in Leccino fruits **(E,G)**; endocarp was almost completely lignified (M). **(D)** is stained with osmium, while the others are stained with toluidine blue.

About mesocarp thickness and cell size, the differences between shade and light fruits were still significant in Leccino and not in Frantoio, but differences between cultivar were not detected (Figure 4A–D and Supplementary Table S1). The interaction between cultivar and light condition was not significantly different for any parameters (Supplementary Table S1). In both cultivars and light conditions, oil drops were uniformly distributed in the mesocarp (Figure 5A–D) and occupied generally a greater fraction of the cell lumen (Figure 5E–H). Differences in size between the oil drops were evident in the fruit semi-thin sections and can be related to a different oil storage. In Leccino, oil drops were larger in light fruits compared to shade ones, while in Frantoio no differences were detected (Figure 4E,F). At this stage and in both light conditions, oil drops appeared to occupy a larger fraction of the cell volume in Frantoio (Figure 5F,H and Supplementary Table S2) than in Leccino (Figure 5E,G and Supplementary Table S2) fruit. Endocarp was almost completely differentiated; the sclerified area was more continuous and the separate cluster of sclereid cells merged (data not shown).

#### 140 DAF

At this stage, no differences were observed about mesocarp thickness between light and shade fruits in Leccino and Frantoio (Figure 4A,B); significant differences were instead observed between cultivar, as Leccino showed a thicker mesocarp than Frantoio (Supplementary Table S1). The mesocarp cell size was, however, larger in light than in shade Leccino fruits (Figure 4C), while no differences were observed between light and shade fruit in Frantoio (Figure 4D). About the cultivar effect, mesocarp cells of Frantoio fruits had a smaller size than those of Leccino fruit (Supplementary Table S1).

**FIGURE 6:**
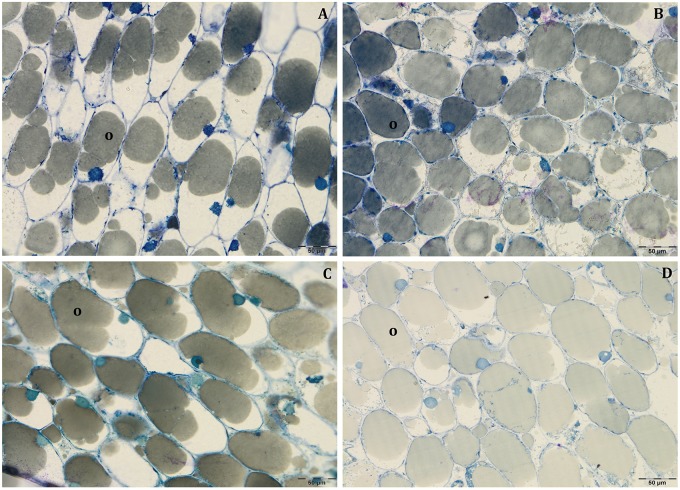
Semi-thin sections at 140 DAF of Leccino **(A,C)** and Frantoio **(B,D)** fruits in light **(A,B)**, and shade **(C,D)** conditions: the cell volume occupied by the oil drops was higher in Frantoio **(B,D)** than Leccino **(A,C)** fruits. All the figures are stained with toluidine blue.

In all collected samples, endocarp was completely lignified, and oil bodies were larger than those detected at 80 DAF (Figure 4E,F). In Leccino, the cell volume occupied by oil drops was considerably lower than Frantoio in both light conditions (Figure 6 and Supplementary Table S2). No differences were observed in oil drops size between shade and light olive fruit.

### Water and Oil Content

At harvest, water and oil composition varied significantly among light exposure (Table 2). In both cultivars shade fruits showed a higher water content than the light ones. The oil content was, instead, higher in the light fruits of both cultivars then in the shade ones. Significant differences were also observed between cultivars in both water and oil content (Supplementary Table S1).

**Table 2:** Water and oil content at harvesting time (20th November).

Cultivar	Exposure	Water content (%)	Oil content (% of fresh weight)	Oil content (% of dry weight)
Leccino	Shade	55.29^a^	18.28^b^	40.80^b^
Leccino	Light	53.81^b^	19.21^a^	41.58^a^
	
Frantoio	Shade	51.66^a^	17.51^b^	36.22^b^
Frantoio	Light	49.90^b^	18.59^a^	37.10^a^


*Light fruits were taken on the external portion of the canopy, while shade fruits were collected in the centre. Different letters indicate statistically significant differences by *T*-test at *p* ≤ 0.05.*

## Discussion

Light availability affects fruit development by a direct effect on carbon metabolism, and indirectly by other mechanisms, such as: (1) the translocation of carbohydrates from nearby reserves, stored in the more lighted portions of plants (Cherbiy-Hoffmann et al., 2013); (2) the photosynthetic activity of the fruit itself that contributes to growth (Proietti and Tombesi, 1996); (3) the increase in fruit temperature, related to higher irradiance exposure, which intensifies the translocation of carbon and other assimilates to the fruit and, therefore, its sink activity (Génard and Bruchou, 1993; García-Inza et al., 2014). The aim of this research was to study the effect of the sunlight on the fruit development of two different olive cultivars, Frantoio and Leccino. Cyto-histological observations carried out in fruits harvested at 14, 21, 50, 80, and 140 DAF, corresponding to the I, II, III, and IV developing phases, respectively (Conde et al., 2008), showed that light irradiance did not influence the succession of these developmental phases: indeed, shade and light fruits in both cultivars did not show differences about the timing of cell differentiation in all fruit tissues. Differences were only observed between cultivars at 21 DAF; indeed, sclereid cells appeared less frequent in Leccino than in Frantoio, suggesting the precocious endocarp lignification in the last one. As proposed also by Trentacoste et al. (2016), the delay in endocarp lignification, observed in Leccino, could produce a greater and longer competition for the assimilates between mesocarp and endocarp, which exacerbate differences related to light availability. In Leccino, the fruit fresh and the dry weight, the percentage of dry matter, the kernel and fruit diameter, the mesocarp thickness, and the mesocarp cell size were higher in the light fruit than the shade ones. In Frantoio, differences between light and shade fruit were observed only at 140 DAF, and only in the kernel and fruit diameter and in the dry and fresh weight, which were higher in the light fruit than the shade ones. Leccino, therefore, showed a greater sensitivity to the light availability, probably related to the delay in the endocarp lignification.

At 140 DAF, which is near to harvesting, the oil content (determined as percentage on dry weight) was different between cultivar and light and shade fruits, while the size of oil drops did not change. These differences in the oil content would be, therefore, related to the number of cells containing oil and not to the oil content in each cell. A different behaviour was observed in the two cultivars; as demonstrated by cyto-histological observations and morphometric measurements, at 140 DAF. In Leccino the ratio between oil drops size and mesocarp cell size was considerably lower than in Frantoio, where oil bodies occupied almost all the cell lumen. Indeed, in Leccino the mesocarp cells size was almost twice that of Frantoio, while oil drops were only 30% larger. The chemical analysis confirmed this observation: the percentage of water and the oil content were higher in Leccino than in Frantoio, but the differences in the water content were greater than those in the oil content. The values relating to the water content of Leccino shade and light fruit were 7.02 and 7.83% higher than those of Frantoio shade and light fruit, respectively; moreover, oil content in Leccino shade and light fruit were 4.39 and 3.33% higher than those of Frantoio shade and light fruits, respectively. In both cultivars, the oil content referred to the fruit dry weight was only slightly higher in light fruits compared to shade ones. However, considering the greater weight of light fruits (Figure 2), the differences in terms of oil content per fruit (g oil/fruit) are higher. In both cultivar, the higher oil content, fresh, and dry weight, fruit and kernel diameter registered in the light fruits compared to the shade ones, were paired to a higher water content in the shade than in the light fruits. These results are in contrast with observations carried out at different water status or with different cultivars, in which larger fruits have higher water content (Motilva et al., 2000; Alegre et al., 2002; Mailer et al., 2007; Martín-Vertedor et al., 2011), but agreed with data collected by Trentacoste et al. (2016). These authors hypothesised that the microenvironment surrounding the olive fruits with different light availability influenced oil accumulation and dry matter composition more strongly than the water accumulation, which drives cell expansion and also fruit growth. The relationships between fruit growth and oil and carbohydrate accumulation are quite complex and were also related to cultivar, as also demonstrated by differences observed in our research between Leccino and Frantoio.

Our data confirmed the influence of light availability on fruit development as previously observed also by other authors (Trentacoste et al., 2016; Caruso et al., 2017), but also outlined that this effect can vary depending on the cultivar. The different behaviour of cultivars could be related to differences observed in the endocarp development; Trentacoste et al. (2016) did not observed any differences in the endocarp size and composition in fruit of the same cultivar in different canopy position, while present data suggested a different endocarp development in Frantoio and Leccino shade and light fruits. Finally, for the first time, to our knowledge, the oil storage in mesocarp cells was investigated by cyto-histological studies, at different light conditions, suggesting that the differences in oil content were not related to a different oil storage in each mesocarp cell. Our data, in fact, demonstrated that oil drops size in each cell was not influenced by light condition.

## Author Contributions

PP, LRea, and FF designed the work. LN collected the samples. LN, MC, PP, and LRea acquired and analysed the data. LRea drafted the manuscript. MC, LN, PP, FF, and LReg critically revised the article. All authors approved the version of the manuscript to be published.

## Conflict of Interest Statement

The authors declare that the research was conducted in the absence of any commercial or financial relationships that could be construed as a potential conflict of interest.
