# Supplementary material for: The Influence of Light on Olive (Olea europaea L.) Fruit Development Is Cultivar Dependent
Source: Front Plant Sci. 2019 Mar 27;10:385. doi: 10.3389/fpls.2019.00385 (PMC6446062; doi:10.3389/fpls.2019.00385)
Supplement: Supplementary file 1 [file Table_1.pdf]

Table S1.ANOVA tables for fresh weight, fruit diameter, dry matter, dry weight, kernel diameter, mesocarp thickness and oil drop size at 50, 80, 140 DAF.

Response: Fresh_weight_140

	Df	Sum Sq	Mean Sq	F value	Pr(>F)	
Light	1	0.8815	0.88153	10.9239	0.001605	**
Cultivar	1	0.1473	0.14728	1.8251	0.181777	
Light:Cultivar	1	0.0087	0.00868	0.1075	0.744122	
Residuals	60	4.8418	0.08070			

Response: Fresh_weight_80

	Df	Sum Sq	Mean Sq	F value	Pr(>F)	
Light	1	1.4674	1.46741	16.0874	0.000181	***
Cultivar	1	0.7567	0.75672	8.2960	0.005620	**
Light:Cultivar	1	0.3537	0.35374	3.8781	0.053875	.
Residuals	56	5.1080	0.09121			

Response: Fresh_weight_50

	Df	Sum Sq	Mean Sq	F value	Pr(>F)	
Light	1	1.12264	1.12264	43.787	5.142e-08	***
Cultivar	1	0.27007	0.27007	10.534	0.002304	**
Light:Cultivar	1	0.67830	0.67830	26.456	6.672e-06	***
Residuals	42	1.07682	0.02564			

Response: Fruit_diameter_140

	Df	Sum Sq	Mean Sq	F value	Pr(>F)	
Light	1	8.446	8.4463	13.3829	0.0005371	***
Cultivar	1	0.464	0.4641	0.7354	0.3945660	
Light:Cultivar	1	0.001	0.0010	0.0015	0.9687530	
Residuals	60	37.868	0.6311			

Response: Fruit_diameter_80

	Df	Sum Sq	Mean Sq	F value	Pr(>F)	
Light	1	15.913	15.9135	22.0269	1.777e-05	***
Cultivar	1	5.281	5.2807	7.3093	0.009067	**
Light:Cultivar	1	2.522	2.5215	3.4902	0.066969	.
Residuals	56	40.458	0.7225			

Response: Fruit_diameter_50

	Df	Sum Sq	Mean Sq	F value	Pr(>F)	
Light	1	12.9169	12.9169	34.674	4.92e-07	***
Cultivar	1	6.2352	6.2352	16.738	0.0001800	***
Light:Cultivar	1	6.6752	6.6752	17.919	0.0001154	***
Residuals	44	16.3908	0.3725			

Response: Dry_matter

	Df	Sum Sq	Mean Sq	F value	Pr(>F)	
Light	1	6.040	6.040	95.794	9.963e-06	***
Cultivar	1	92.585	92.585	1468.450	2.362e-10	***
Light:Cultivar	1	2.327	2.327	36.911	0.0002974	***
Residuals	8	0.504	0.063			

Response: Dry_weight

	Df	Sum Sq	Mean Sq	F value	Pr(>F)	
Light	1	0.0212483	0.0212483	23.8390	0.00122	**
Cultivar	1	0.0060106	0.0060106	6.7435	0.03177	*

	Df	Sum Sq	Mean Sq	F value	Pr(>F)	
Light:Cultivar	1	0.0025534	0.0025534	2.8647	0.12900	
Residuals	8	0.0071306	0.0008913			

Response: Kernel_diameter_140DAF

	Df	Sum Sq	Mean Sq	F value	Pr(>F)	
Light	1	5.0603	5.0603	23.2498	9.142e-06	***
Cultivar	1	0.1096	0.1096	0.5036	0.4805	
Light:Cultivar	1	0.1478	0.1478	0.6790	0.4130	
Residuals	64	13.9297	0.2177			

Response: Kernel_diameter_80DAF

	Df	Sum Sq	Mean Sq	F value	Pr(>F)	
Light	1	1.3635	1.36350	2.9678	0.09196	.
Cultivar	1	2.8373	2.83727	6.1757	0.01683	*
Light:Cultivar	1	0.4089	0.40885	0.8899	0.35065	
Residuals	44	20.2147	0.45942			

Response: Kernel_diameter_50DAF

	Df	Sum Sq	Mean Sq	F value	Pr(>F)	
Light	1	4.1615	4.1615	8.2906	0.006669	**
Cultivar	1	0.3877	0.3877	0.7724	0.385315	
Light:Cultivar	1	0.7868	0.7868	1.5675	0.218649	
Residuals	36	18.0705	0.5020			

Response: Mesocarp_thickness_140DAF

	Df	Sum Sq	Mean Sq	F value	Pr(>F)	
Light	1	1116	1116	0.0227	0.8826564	
Cultivar	1	1352929	1352929	27.4586	0.0001596	***
Light:Cultivar	1	20148	20148	0.4089	0.5336254	
Residuals	13	640531	49272			

Response: Mesocarp_thickness_80DAF

	Df	Sum Sq	Mean Sq	F value	Pr(>F)	
Light	1	461127	461127	9.3146	0.003384	**
Cultivar	1	39071	39071	0.7892	0.377881	
Light:Cultivar	1	232944	232944	4.7054	0.034041	*
Residuals	60	2970342	49506			

Response: Mesocarp_thickness_50DAF

	Df	Sum Sq	Mean Sq	F value	Pr(>F)	
Light	1	418728	418728	11.359	0.0014717	**
Cultivar	1	1116349	1116349	30.282	1.351e-06	***
Light:Cultivar	1	588650	588650	15.968	0.0002162	***
Residuals	49	1806364	36865			

Response: Oil_drops_140DAF

	Df	Sum Sq	Mean Sq	F value	Pr(>F)	
Light	1	1274485	1274485	1.3266	0.2505917	
Cultivar	1	14073741	14073741	14.6492	0.0001664	***
Light:Cultivar	1	35960	35960	0.0374	0.8467598	
Residuals	233	223846952	960717			

Response: Oil_drops_80

	Df	Sum Sq	Mean Sq	F value	Pr(>F)	
Light	1	2330789	2330789	15.428	0.000107	***
Cultivar	1	2471729	2471729	16.361	6.706e-05	***

Light:Cultivar	1	7286400	7286400	48.230	2.464e-11	***
Residuals	292	44113822	151075			

Response: Oil_drops_50

	Df	Sum Sq	Mean Sq	F value	Pr(>F)	
Light	1	81212	81212	1.8462	0.1758	
Cultivar	1	109127	109127	2.4808	0.1169	
Light:Cultivar	1	1504786	1504786	34.2089	2.058e-08	***
Residuals	195	8577693	43988			

Response: Cell_size_140

	Df	Sum Sq	Mean Sq	F value	Pr(>F)	
Light	1	39927323	39927323	14.1772	0.0002035	***
Cultivar	1	306453219	306453219	108.8138	< 2.2e-16	***
Light:Cultivar	1	13949494	13949494	4.9531	0.0268547	*
Residuals	275	774484980	2816309			

Response: Cell_size _80

	Df	Sum Sq	Mean Sq	F value	Pr(>F)	
Light	1	14313392	14313392	7.2798	0.007303	**
Cultivar	1	229898	229898	0.1169	0.732592	
Light:Cultivar	1	2988436	2988436	1.5199	0.218437	
Residuals	359	705854187	1966168			

Response: Cell_size_50

	Df	Sum Sq	Mean Sq	F value	Pr(>F)	
Light	1	28669999	28669999	23.282	1.757e-06	***
Cultivar	1	25355383	25355383	20.591	6.814e-06	***
Light:Cultivar	1	25490053	25490053	20.700	6.447e-06	***
Residuals	629	774548896	1231397			

Response: Water_content

	Df	Sum Sq	Mean Sq	F value	Pr(>F)	
Light	1	7.857	7.857	54.3832	7.808e-05	***
Cultivar	1	42.676	42.676	295.3896	1.336e-07	***
Light:Cultivar	1	25490053	25490053	20.700	6.47e-06	***
Residuals	8	1.156	0.144			

Response: Oil_content

	Df	Sum Sq	Mean Sq	F value	Pr(>F)	
Light	1	3.07041	3.07041	110.0176	5.937e-06	***
Cultivar	1	1.44908	1.44908	51.9227	9.193e-05	***
Light:Cultivar	1	0.01688	0.01688	0.6047	0.4592	
Residuals	8	0.22327	0.02791			
