# Supplementary material for: The Influence of Light on Olive (Olea europaea L.) Fruit Development Is Cultivar Dependent
Source: Front Plant Sci. 2019 Mar 27;10:385. doi: 10.3389/fpls.2019.00385 (PMC6446062; doi:10.3389/fpls.2019.00385)
Supplement: Supplementary file 2 [file Table_2.pdf]

Table S2. ANOVA table and t-test for oil percentage at 80 and 140 DAF.

ANOVA

Response: % Oil_80

```
                 Df Sum Sq Mean Sq F value    Pr(>F)
Light             1  504.6   504.6  6.3144   0.01336 *
Cultivar          1 6793.1  6793.1 85.0139 1.701e-15 ***
Light:cultivar  1  130.2   130.2  1.6297   0.20431
Residuals       115 9189.2    79.9
```

Response: % Oil_140

```
                 Df  Sum Sq Mean Sq  F value  Pr(>F)
Light             1   132.6   132.6   1.1280 0.29042
Cultivar          1 14192.8 14192.8 120.7133 < 2e-16 ***
Light:cultivar     1   795.2   795.2   6.7636 0.01053 *
Residuals       115 13521.1   117.6
```

T-test

	Frantoio light 80 DAF	*Leccino light 80 DAF*
Mean	42.4533	25.23098
Variance	185.4508	35.40671
Stat t	6.257461	
P(T<=t)	2.53E-07	
t critical	2.024394	

	Frantoio shade 80 DAF	*Leccino shade 80 DAF*
Mean	44.33332	31.29585
Variance	67.01687	35.3892
Stat t	7.056525	
P(T<=t)	3.66E-09	
t critical	2.005746	

	Frantoio light 140 DAF	*Leccino light 140DAF*
Mean	77.07678	55.84766
Variance	87.03893	82.19466
Stat t	8.859966	
P(T<=t)	2.62E-12	
t critical	2.002465	

	Frantoio shade 140 DAF	*Leccino shade 140 DAF*
Mean	79.6175	62.90053
Variance	167.6522	95.12487
Stat t	5.648389	
P(T<=t)	6.21E-07	
t critical	2.004879	
